# Measles Outbreak in Africa—Is There a Link to the HIV-1 Epidemic?

**DOI:** 10.1371/journal.ppat.1001241

**Published:** 2011-02-10

**Authors:** Anna Nilsson, Francesca Chiodi

**Affiliations:** 1 Department of Women and Child Health at Karolinska Institutet, Astrid Lindgrens Childrens Hospital, Stockholm, Sweden; 2 Department of Microbiology, Tumour and Cell Biology at Karolinska Institutet, Stockholm, Sweden; The Fox Chase Cancer Center, United States of America

Measles remains an important cause of child mortality, although the numbers of measles-related deaths has decreased during the last decade [1] through childhood immunisation programmes and follow-up measles vaccine campaigns. In 2005, the World Health Organization (WHO) and the United Nations Children's Fund (UNICEF) launched a global plan to further reduce measles mortality in the years 2006–2010 [2]. Despite these joint efforts, an increased number of large and deadly outbreaks of measles on the African continent were reported, with the most severe outbreaks in Chad, Nigeria, and Zimbabwe. The current increase in measles cases has been attributed to a failure in maintaining high measles vaccine coverage [3]. There are obviously several factors of medical and social relevance to take into consideration when trying to explain the increased measles outbreaks in Africa. In this article, our focus is to highlight the possibility of a co-existing link between the measles outbreaks and pathological features of HIV-1 infection in mothers and children, as the measles outbreaks occurred in countries with a high HIV-1 prevalence.

Passively acquired maternal antibodies protect infants against measles until the time of measles vaccination, which in most developing countries is administered at 9 months of age. In 1992, an increased risk of measles before 9 months of age was reported in children born to mothers with HIV-1 [4], which was suggested to be due to lower levels of passively acquired antibodies at birth [5]. In a recent study, the level of measles antibodies were followed from 6 weeks of age until 11 months in HIV-1-infected, HIV-1-exposed non-infected (born to mothers with HIV-1 but not HIV-1 positive), and HIV-1-seronegative children [6]. By 6 months of age, 91% and 83% of HIV-1-infected and HIV-1-exposed non-infected children had measles antibody levels of <50 mIU/mL (cut-off value for specific immune response); 42% of HIV-1-negative children, on the other hand, retained high antibody levels at 6 months. These findings confirm the previous observation [5] of low titres of maternal antibodies being transferred to infants of mothers with HIV-1. Children born to mothers with HIV-1 have a higher risk of contracting early measles independently of whether they are themselves HIV-1 infected [5]. In a study from Zambia, co-infection with HIV-1 and measles in children was shown to more than double the risk of death in measles during hospitalisation [7]. Deaths due to measles infection occurred in 12.2% of the children with HIV-1 (median age 12 months) as compared to 4.3% of non-HIV-1-infected children (14 months). Since the control of measles and HIV-1 relay on efficient CD8 T cell responses, the increased morbidity observed in children with HIV-1 upon measles infection can be related to the shift in cytokine profile from Th1 to Th2 occurring in these young individuals and impairing T cell responses to both pathogens [8]. A Th1 to Th2 shift during the course of chronic HIV-1 infection is associated with progression to AIDS [9], and measles virus infection also suppresses the ability of T cells to produce IL-12, thus hampering T cell responses [10].

To reduce the risk of contracting measles in areas with high HIV-1 prevalence, WHO recommended that infants receive two doses of measles vaccine, at 6 and 9 months [11]. This regimen was evaluated in Zambia [12] and results published in 2008 showed that 59% of children with HIV-1 were measles antibody positive after the first vaccine dose; this number increased to 64% after the second dose. Among HIV-1-exposed non-infected children, 68% and 94% were seropositive after the first and second immunisation, respectively, and similar figures were shown for control children (62% and 92%). To further pinpoint the B cell impairments leading to low antibody levels after measles vaccination in children with HIV-1, Nair [13] characterised early antibody responses to measles following vaccination at 9 months of age. Interestingly, HIV-1 infection impaired IgG responses after vaccination as well as the development of high avidity measles antibodies. In a study from Kenya, antibody titres to measles were evaluated 2 to 5 years after measles immunisation received during the first year of life [14]. Several years after immunisation, only 33% of the children with HIV-1 maintained measles IgG antibodies, indicating impairment in the establishment and the maintenance of serological memory responses.

Which, then, could be the mechanism accounting for the decreased amount of measles antibodies circulating in mothers with HIV-1 and poor response to measles vaccination in children with HIV-1? The rapid IgM immune response towards measles occurring to a great extent in the splenic marginal zone B cells is an important first-line defence upon natural infection and after immunisation. In healthy children, the splenic marginal zone is not fully developed until 2 years of age [15], and this observation explains the low responses observed upon vaccination in young children. During HIV-1 infection, the structure of lymphoid tissue is altered, leading to follicular hyperplasia and likely to impairment of marginal zone responses [16].

**Figure 1:**
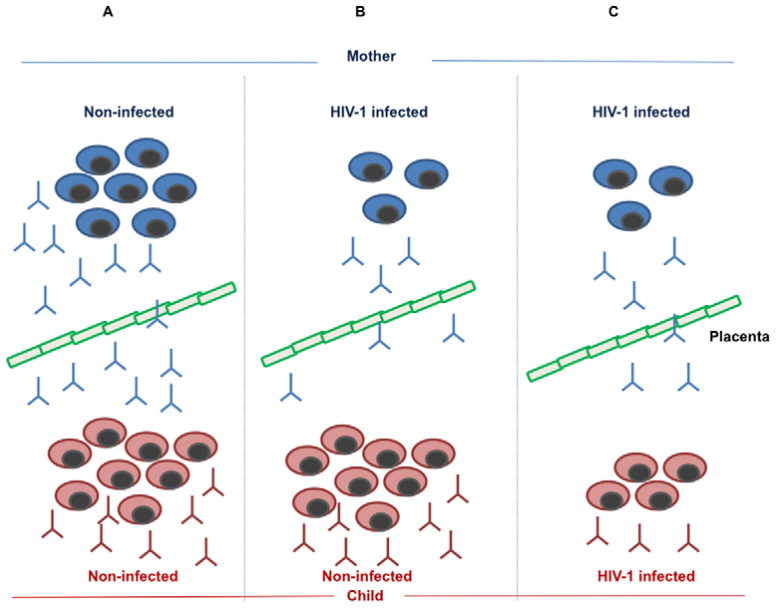
Maintenance and formation of measles-specific antibodies and memory B cells in mother and child. (A) In the non-infected mother, a normal number of memory B cells produce a protective level of measles specific antibodies, which are transmitted to the child via the placental barrier. In addition, the child will respond to measles vaccination by producing memory B cells and specific antibodies. (B) The HIV-1-infected mother loses a large number of memory B cells as a result of pathogenic mechanisms linked to HIV-1 infection; this phenomenon leads to a reduced amount of measles-specific antibodies in the mother and a low level of transmitted antibodies to the child. The HIV-1-exposed, non-infected child, is, however, competent to respond to measles vaccination by generating protective levels of measles antibodies and measles-specific memory B cells. (C) As a consequence of HIV-1 infection, both the HIV-1-infected mother and child lose measles-specific memory B cells formed upon measles natural infection or vaccination. This leads to a low, non-protective level of measles antibodies in the mother, a low level of antibodies transmitted through the placental barrier to the child, and a low, non-protective level of measles-specific antibodies produced from the infected child upon vaccination.

We suggest that the decline of resting memory B cells reported by us [17]–[19] and others [20] that occurs during HIV-1 infection may be an important pathogenic mechanism linked to the low level of measles-specific antibodies found in mothers with HIV-1 and their children (Figure 1). Memory B cells are responsible for mounting and maintaining an adequate serological response to antigens previously encountered in life through natural infection or vaccination. The decline in B cells carrying immunological memory correlated to loss of antibody titres to measles, tetanus, and pneumococcal antigens [17], [18]. Interestingly, in turn, the decline of serum measles antibodies correlated to a decreased number of measles-specific memory B cells in blood. The antibody levels to pneumococcal antigens were dramatically reduced already from primary HIV-1 infection [17].

Our studies on the loss of memory B cells strongly suggest that this pathogenic mechanism may be causing a reduced level of protective anti-measles antibodies in mothers with HIV-1; it is also very likely that the levels of measles antibodies in breast milk of mothers with HIV-1 may be reduced in comparison to that of healthy women. The loss of memory B cells was positively correlated to the number of CD4+ T cells, a pivotal hallmark of immune deficiency during HIV-1 infection [17]. It is likely that an increased number of CD4+ T cells following highly active antiretroviral therapy (HAART) may lead to a new generation of measles-specific memory B cells through repeated antigenic exposure in countries with high measles prevalence.

Active HIV-1 replication correlates to the lack of development of an adequate serological memory as measured by the poor response to measles vaccination occurring in children with HIV-1 [21]. We showed that HAART treatment administered early after birth led to control of HIV-1 replication and also preserved the evolution of the memory B cell compartment and the likelihood of response to childhood vaccines. In children treated after 1 year of age, a decline in memory B cells was observed, accompanied by a modest response to measles vaccination. When matched with the measles vaccination studies conducted in developing countries, our findings indicate that a low level of protective measles antibodies in children with HIV-1, resulting from the impaired incapacity to mount serological memory, may represent the cause of measles outbreaks in countries with high levels of HIV-1 infection.

It is encouraging that measles catch-up vaccination programmes have been shown to reduce measles morbidity and mortality in southern Africa, although children born to mothers with HIV-1 remained highly susceptible to measles infection and its lethal consequences [22]. In conclusion, the recommended vaccination schedule to eradicate measles may be inadequate in countries with a high proportion of adults and children with HIV-1. According to the findings presented in this article, we propose that HAART should be administered to children and adults with HIV-1 prior to measles vaccination since HAART improves the capacity to establish long-term serological memory and maintain memory B cell responses in individuals with HIV-1.
